# Russia’s Hybrid Warfare in Ukraine Threatens Both Healthcare & Health Protections Provided by International Law

**DOI:** 10.5334/aogh.4022

**Published:** 2023-01-23

**Authors:** Michael S. Baker, Jacob Baker, Frederick M. Burkle

**Affiliations:** 1 Rear Admiral, US Navy (Ret), US; 2 Merit International, Inc., US; 3 Woodrow Wilson International Center for Scholars, Washington, DC, US

**Keywords:** Hybrid Warfare, hospital attacks, critical infrastructure, civilian attacks, war crimes

## Abstract

Hybrid Warfare is on display because of the unjustified Russian invasion of Ukraine. This is characterized by numerous crimes against civilians as seen vividly during the occupation of the town of Bucha where rape, torture, murder, and looting seem to reflect Russian military policy, leadership, and command guidance. Of particular concern is the threat to hospitals and health care as well as vital life support. Numerous hospitals have been damaged and destroyed. Hospitals are not tactical military targets and targeting health care facilities and personnel ignores traditional jus in bello and ignores numerous conventions established to stabilize the global order.

The Russian-proclaimed “special operation” in Ukraine has been characterized by barbarian warfare in which the Russian military uses weapons against the civilian population and civilian infrastructure. The aggressors have embarked on a purposeful terror campaign through infrastructure attacks, which are of little military value except to demoralize the nation’s people. This is evident with Russian missile and drone attacks on electric, water, and health care in Ukraine.

Warfare now and in the future may be increasingly aimed at demoralizing civilian populations and reducing the will of the people and their government to resist. The Ukrainian invasion clearly shows that this use of hybrid warfare should be met with a strong reaction of the international community at the earliest possible stage, especially the supposedly peace-loving neutral countries, or else the future is expanded unlawful and barbaric military conflict.

## Current Threat Environment

The rise in friction between nation states due to numerous global conflicts and the risk of state–on–state confrontation increasingly confronts the US, its military, and their allied partners – especially considering the outbreak of war in Europe with the Russian invasion of Ukraine. As such, widespread conflagration could expand in the future due to a number of reasons: the conflict between Russia and Ukraine, Great Power competition in the Arctic, attacks on communications cables in the Baltic Sea, aggressive hegemonic actions in the South China Sea, or continued provocations from North Korea. Terrorist, criminal, and other non–state actors threaten global security, and international law conventions protecting healthcare facilities and workers are at increased risk.

Russia has previously invaded Crimea (Ukraine), Transdniestria (Moldova), South Ossetia, and Abkhazia (Georgia). Russia had also stoked two non–international armed conflicts (NIACs) in Ukraine, opposing governmental forces with the self–proclaimed “People’s Republics’ of Donetsk and Luhansk in Eastern Ukraine” [1]. Europe is now the theatre of an international armed conflict (IAC) between Ukraine and Russia, a conflict that could easily spill over and ignite major clashes in neighboring countries. Russian previous actions in Syria and Chechnya, as well as the above countries demonstrate what appears to be a strategy of destroying infrastructure and demoralizing the local populations.

It is commonplace for Ukrainian soldiers and international volunteers to refer to invading Russian soldiers as “orcs”, characterized as “a brutish, aggressive, ugly, and malevolent race of monsters” that have been let loose to terrorize peaceful, democratic people in Central Europe [2]. This description encompasses an unjustified invasion characterized by the numerous crimes against civilians. This was seen vividly during the occupation of the town of Bucha, where rape, torture, murder, and looting seemed to reflect guidance from leadership and command [3]. Of particular concern is the threat to hospitals and healthcare, along with vital life support, as we see the aggressors embarking on a purposeful terror campaign through attacks on infrastructure. Warfare now and in the future may be increasingly aimed at demoralizing civilian populations – eroding at the government and citizens’ will to resist. This is evident with the Russian missile and drone attacks on electric, water, and healthcare in Ukraine.

Russia seemingly ignores international norms by disregarding the 1994 Budapest Security Agreement and the United Nations’ guidelines against force by brazenly invading another sovereign nation. Russia’s annexation of Crimea and its prior covert invasion of Eastern Ukraine places uncomfortable doubt on the worth of existing security assurances pledged to Ukraine by the nuclear powers in exchange for Ukrainian denuclearization. In 1994, the three signatory states completed an agreement known as the “Budapest Memorandum”, which provided positive security assurances for Ukraine [4]. The depository states underlined their commitment to Ukraine’s sovereignty and territorial integrity by signing the memorandum, in exchange for Ukraine giving up its nuclear weapons. The failure of the Budapest Memorandum to deter one of Ukraine’s security guarantors from military aggression has important implications both for Ukraine’s long–term security, and for the value of security assurances for future international nonproliferation, disarmament efforts, and peaceful coexistence.

Twenty–first century warfare is exemplified by multi–domain operations, asymmetry, and a hybrid approach. Hybrid warfare entails an interplay or fusion of conventional as well as unconventional instruments of power and tools of subversion. These instruments or tools are blended in a synchronized manner to exploit the vulnerabilities of an antagonist and achieve synergistic effects. Russia initiated a “hybrid war” that uses all components of hybrid warfare, including “political, diplomatic, economic, and financial warfare, legal (law–fare)”, as well as socio–cultural efforts, with infrastructure, intelligence, and criminal groups also widely used [5]. The target of warfighting in hybrid warfare is not limited to the military and is expanded to include civilians by creating political instability through cyberattacks, disinformation, and the disruption of daily lives. Attacks on civilian life support and infrastructure that has little obvious immediate military value are rampant in Russia’s strategy to crush Ukrainian resistance.

The armed conflict in Ukraine has fostered an expansion of visible warfare abuses on the civilian population. This is evident in the attacks on infrastructure, as well as in the war crimes against the civilian population perpetrated by the invading Russian soldiers. In all wars, healthcare infrastructure and personnel are at some risk. Since the full–scale invasion of Ukraine on February 24, 2022, the Ukrainian Ministry of Health has reported that the Russians have damaged 788 medical facilities and “turned another 123 into piles of stones” [6]. The Red Cross symbol is seemingly a target for non–state actors or unprincipled adversaries. This was evident in the Russian military attacks on hospitals, emergency responders, and health workers [7]. One must question whether this will also extend to Geneva Convention–protected hospitals or hospital ships in the future [8].

It is crucial to both assess the risks and work to ensure the safety of healthcare personnel, especially considering the Russian strategy of targeting civilian facilities and life support systems, with purposeful attacks on infrastructure, healthcare facilities, and personnel. Under the guise of unilateral and supposed peacekeeping operations in neighboring Moldova, Abkhazia, South Ossetia, Nagorno–Karabakh, Syria, and Chechnya, the Russians were anything but impartial [9]. Russia, under their national peacekeeping flag – which did not represent a UN sanctioned operation – showed a callous disregard for civilian life by deliberately targeting civilian infrastructure, with unlawful attacks on hospitals, schools, and civilian gatherings [9]. Similar abuses were documented in Chechnya and Syria, where extreme and shocking violations of human rights and deliberate attacks on the civilian population, public transport, and health workers have been recorded [10]. The International Federation of Human Rights (FIDH) concluded that these violations “constituted war crimes and crimes against humanity due to their massive, systematic, and generalized character, according to the definitions of customary international law, such as the 1977 Additional Geneva Protocols and the different international bodies” [11,12].

Russia continued their strategic application of intense civilian attacks to vital economic infrastructure, healthcare facilities, and civilian populations with little criticism from the West during their invasion of Ukraine [13]. Hybrid warfare and attacks on healthcare facilities should generate strong reactions from the international community with the investigation and publication of studies emphasizing these unlawful attacks on the civilian population [1,9]. The focus on civilian and healthcare targets is currently owned and visibly promoted by Russian policies, its leadership, and their soldiers’ actions [14]. As such, the lack of negative consequences for Russia and its leaders will embolden other aggressors to promote similar strategies aiming to destroy morale through the purposeful targeting of healthcare facilities, both civilian and military, in any future conflicts.

Current healthcare facility–related abuses represent grave violations of the Geneva Convention and are subject to “universal jurisdiction” under international law [15]. Russia has previously and has continued to show blatant disdain for existing international humanitarian law, the Geneva Conventions, and human rights law since their 2019 withdrawal from Article 90 of Protocol I of the Geneva Convention. They must feel that this exception protects themselves from any prosecutions in the future, especially for the aggressions perpetrated against Ukraine. This includes abuses related to healthcare, such as the destruction of hospitals and other health facilities. No other country enjoys such provisions, although one should expect such claims to arise from Russian allies and perhaps other aggressor nation states in the future [16,17]. Thus far, very few Russian war crime perpetrators have been called to account, and these have been in Ukrainian courts for common soldiers.

The International Criminal Court (ICC) announced its jurisdiction over potential war crimes in Ukraine, relying on recent requests from the Ukrainian government. Governments of 39 signatory states to the Rome Statute, which created the ICC, also submitted formal requests for the ICC’s jurisdiction in this instance. Prosecution for war crimes and reparations are complex and will probably take years to complete. Bringing the perpetrators and the leaders who promoted those crimes to an international court is very difficult because any efforts to issue arrest warrants against Russian nationals would be futile, given that the ICC relies heavily on national authorities to apprehend suspects and transfer them to The Hague for trial. This makes prosecution unlikely unless regime changes in Russia takes place [18].

Today’s global commons have become a much more unstable and risky place with accelerating change from global warming, widespread famines, pandemic outbreaks, economic collapse, ethnic conflicts, transnational organized crime, and rising levels of nationalism–fueled friction. The destruction of Geneva Convention–protected hospitals or ships by non–state actors or unprincipled adversaries – who often target the Red Cross symbol specifically – can adversely affect the morale of both the civilian population and fighting forces by removing access to healthcare. Unarmed, soft targets invite increased aggression in our current milieu.

One should assume that other future adversaries will be similarly attacking civilians. The potential for state–sponsored conflicts includes using armed civilian proxies, self–defense militias, imported paramilitary units (such as the Russian supported Wagner Group of mercenaries), and threats such as piracy and transnational crime. These present a far different paradigm for the US and its allies to confront [18,19,20]. A terrorist team, paramilitary group, or criminal enterprise, whether composed of non–state actors or clandestine operatives of sovereign entities, will likely neither acknowledge nor support conventions to protect non–combatants. They might intentionally target non–combatant personnel, facilities, and activities, both civilian and military – knowing that their actions would not lead to global legal consequences. They might prefer unprotected platforms and people as targets, both for their vulnerability and the shock value of their destruction. Outdated rules of conventional and customary international law, which were designed for a bygone era, will hamper international humanitarian missions and imperil the safety of hospitals and personnel. The United Nations is not silent on this issue, but is somewhat powerless to intervene. “Neutral” countries implicitly enable and perhaps support these attacks on civilians, infrastructure, and healthcare facilities through their silence and economic cooperation with Russia [21].

## Conclusion

Hospitals displaying the protective symbol of the Red Cross, Red Crescent, or similar medical insignia have a moral majesty about them that demonstrates our shared humanity. These platforms and their personnel convey the message that even in the depths of destruction and despair that so often accompany armed conflicts or natural disasters, “help and hope” is present. That is a crucial pillar of our civilization.

Today’s complex geopolitics in conjunction with rapid global change have rendered prior international legal conventions outdated. The current experience demonstrates Russia’s policy abuses on both a governmental and military level. Its conduct in the Russian invasion of Ukraine violates international humanitarian law, the Geneva Conventions, and the long–standing tradition of protecting medical facilities and personnel [22]. This along with the history of horrific hybrid war abuses focused on the civilian population and healthcare facilities in Syria and Chechnya clearly places all hospital facilities and healthcare personnel at increased risk of becoming tactical targets. The authors strongly recommend that an increased focus on opposing these practices becomes an immediate global top priority. Investigating and spotlighting these transgressions should then lead to consequences for the perpetrators – individually and collectively. Allied governments and the United Nations must step up to restore norms through diplomacy, economic, and legal pressure. The future of healthcare facilities and personnel given the current unabated and unprecedented violations of international law is in open and unprecedented jeopardy.

The UN should step up and remove Russia from the Security Council or suspend its veto power because of its conflict of interest. The presence of Russia on the Security Council with a veto – whilst invading a sovereign nation – is in contravention of the UN Charter. Civilized nations must push back against these abuses and punish these transgressions now, or silently adapt to the global reality of Russia’s demonstrated attacks on civilians, its cruelty, and its war crimes. Clearly there must be a change to the UN Charter to account for unprincipled and illegal activities being carried out by a Security Council member with veto power. It is also time for “neutral” countries to stop buying cheap Russian oil, which helps Russia finance and further its hegemonic ambitions, thereby promoting their cruelties against neighboring populations. Trade must be curtailed to diminish funding for the Russian war and limit the import of material to sustain Russian military forces, and along with other sanctions on Russian banks, companies and individuals need to sting until Russian withdraws from Ukraine. Everyone everywhere is at risk and doing nothing invites further transgressions from transgressive actors.
